# Characterizing internal models of the visual environment

**DOI:** 10.1098/rspb.2025.0602

**Published:** 2025-08-20

**Authors:** Micha Engeser, Susan Ajith, Ilker Duymaz, Gongting Wang, Matthew J Foxwell, Radoslaw M Cichy, David Pitcher, Daniel Kaiser

**Affiliations:** ^1^ Department of Mathematics and Computer Science, Physics, Geography, Justus Liebig University Giessen, Giessen, HE, Germany; ^2^ Center for Mind, Brain and Behavior, Universities of Giessen, Marburg, and Darmstadt, Marburg, HE, Germany; ^3^ Department of Education and Psychology, Freie Universität Berlin, Berlin, Germany; ^4^ Department of Psychology, University of York, York, UK; ^5^ Cluster of Excellence "The Adaptive Mind", Universities of Giessen, Marburg, and Darmstadt, Giessen, HE, Germany

**Keywords:** visual perception, scene representation, predictive processing, internal models, individual differences, drawings

## Abstract

Despite the complexity of real-world environments, natural vision is seamlessly efficient. To explain this efficiency, researchers often use predictive processing frameworks, in which perceptual efficiency is determined by the match between the visual input and internal models of what the world should look like. In scene vision, predictions derived from our internal models of a scene should play a particularly important role, given the highly reliable statistical structure of our environment. Despite their importance for scene perception, we still do not fully understand what is contained in our internal models of the environment. Here, we highlight that the current literature disproportionately focuses on an experimental approach that tries to infer the contents of internal models from arbitrary, experimenter-driven manipulations in stimulus characteristics. To make progress, additional participant-driven approaches are needed, focusing on participants’ descriptions of what constitutes a typical scene. We discuss how recent studies on memory and perception used methods like line drawings to characterize internal representations in unconstrained ways and on the level of individual participants. These emerging methods show that it is now time to also study natural scene perception from a different angle—starting with a characterization of an individual’s expectations about the world.

## 1. Natural vision and internal models of the world

Perceptual efficiency is often understood through the lens of predictive processing [1,2]. In this framework, visual inputs are routinely compared against internal models, which are based on our expectations of what the world should look like. In the processing of natural environments, internal models should play a particularly helpful role [3,4]: natural scenes are reliably structured, with a global structure that is stable across instances of a category and objects placed in statistically predictable locations [5–10]. The reliable structure of natural scenes should give rise to rich internal models that capture what a specific scene (e.g. a kitchen) should typically look like.

The study of vision as an inverse inference problem has its origins in Helmholtz’s idea of perception [11]. Within this framework, the perceptual system uses prior knowledge about the world, obtained through experience, to infer the causes of proximal stimulus patterns. In this view, internal models of the world, which contain this prior knowledge, are thus critical determinants for further efficient natural perception. This was later highlighted by schema theory, which postulated that inputs are referenced against internal models (schemata) that reflect the structure of the world (e.g. the likely object arrangements found in a scene; [12,13]). This concept has influenced early research on scene perception [14,15] and memory [16,17]. In contemporary research, the idea reverberates in the use of predictive processing frameworks for explaining how we perceive [2,18,19] and explore natural scenes [20] as well as how they are analysed in the brain [21,22].

Together, the currently favoured theoretical frameworks converge towards a view in which the contents of our internal models shape how we perceive the world. Yet, critical questions remain unsolved: what exactly are the contents of the internal models that guide natural vision? And, given the variability in visual experiences, how do these models differ across individuals? Here, we discuss how internal models have classically been characterized in the domain of natural scene perception. Reviewing this literature, we distil a need for complementary methods that enable individual participants to report the characteristics of their internal model of a scene. We highlight emerging methods suitable for this purpose, including drawings, scene arrangements, linguistic descriptions and neuroimaging-based methods.

## 2. The classical approach to characterizing internal models

### (a) Probing internal models of scenes by manipulating input characteristics

**Figure 1.**
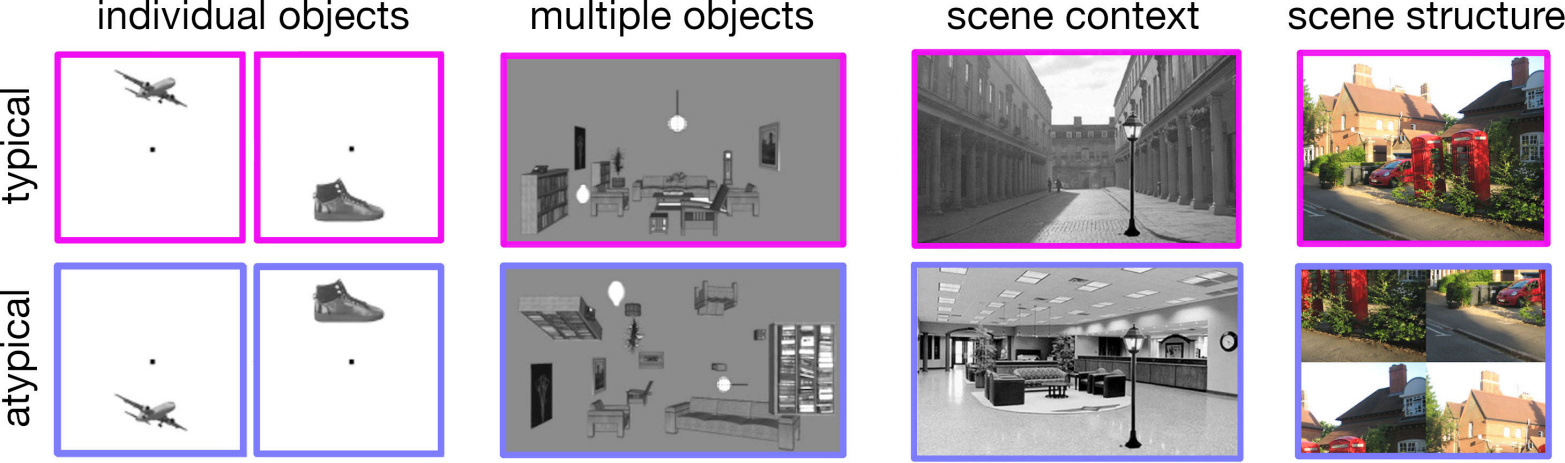
Understanding internal models through stimulus manipulation. To infer the contents of internal models of the world, researchers have manipulated the real-world typicality of visual inputs on different levels. From left to right: manipulations in the typical positioning of individual objects across visual space [23], the typical composition of multiple objects across space (figure is reproduced from [24]), the semantic consistency between scenes and the objects they contain [25] and the structural coherence of the scene [26]. By comparing typically arranged stimuli with atypically arranged stimuli, such studies show that the visual system preferentially processes stimuli that are in accordance with our priors.

Previous work was built on the assumption that the contents of internal models can be studied by varying the level of typicality (i.e. schema-congruence) in the input. Under this assumption, internal models can be characterized by studying responses to stimuli that are in accordance with or in conflict with typical real-world experience, and thus with internal models (figure 1). Through such manipulations, researchers were able to isolate various aspects of typical scene structure that facilitate perception and cortical processing. These include the positioning of individual objects across space [23,27,28], spatial relationships between objects [24,29–32], contextual relationships between scenes and objects [33–36] and the spatial configuration of the scene as a whole [14,37–39]. Together, these studies show that typical scene structure at multiple levels of description contributes to the efficient perception and neural representation of scenes (for reviews, see [5–10,19]), suggesting that internal models contain rich information about the typical properties of natural scenes.

### (b) Challenges for the stimulus manipulation approach

While the approach of manipulating stimulus characteristics has led to significant advances in our understanding of the contents of our internal models of the world, this ‘classical’ approach has several critical downsides.

First, it largely rests on the experimenter’s intuition of what a typical scene looks like and which factors construe its typicality. Although researchers have started using computational analyses to determine typical scene properties more objectively, for example, by extracting object distributions across large scene databases [6,40,41], such approaches are still centred on the idea that the property selected by the researcher plays an important role. Properties that intuitively should be featured in our internal models because they are visually prominent (e.g. the colour of the objects contained in a bathroom versus living room) can, in principle, be relatively uncritical for scene processing, while others that are harder to grasp intuitively (e.g. the relative orientation of objects towards each other) may be exploited by the brain more strongly.

Second, stimulus manipulation approaches only allow for independently manipulating particular stimulus dimensions at a time. Natural scenes, however, cannot be easily decomposed into a few orthogonal dimensions. Furthermore, the relevant dimensions probably interact with each other: in a kitchen scene, objects like an oven or sink are typically aligned along the walls, whereas smaller objects like utensils or cups are placed on horizontal surfaces. Studies separately investigating object distributions or scene geometry may therefore miss critical interactions between these properties. On a practical end, studies that look at such different factors tend to employ different experimental paradigms, making it hard to compare their relative contributions.

Third, many studies use artificial or unusual stimuli to create ‘atypical’ scenes. For instance, in studies of scene–object congruence [25,33,34], researchers need to create incongruent conditions, in which the objects are positioned atypically: a living room lamp is shown on a street, and the streetlight in a living room. Recent behavioural work suggests that differences between congruent and incongruent conditions may indeed be driven by a ‘congruency cost’, where the unexpected, incongruent objects gain a relative processing advantage [42]. Moreover, on the cortical level, spatially distinct regions code for congruent and incongruent conditions [43]. The problem is further aggravated when the atypical conditions violate the laws of physics [24,31] or produce unnaturalistic inputs [7].

Finally, the stimulus manipulation approach neglects inter-individual differences in internal models. Yet, internal models probably differ across individuals: although a typical kitchen will probably look somewhat similar for two people, there may be critical differences, for instance, in the placement of objects. Such individual differences may relate to idiosyncratic visual diets, but also to cultural, linguistic or socioeconomic factors [44,45]. If we could harness this variability, we may find that there are characteristic differences in the way each of us perceives the world, based on our idiosyncratic priors of what the world looks like.

To overcome these critical differences, an approach focused on obtaining descriptions of internal models directly from the participants bears enormous potential. Moving on, it is important to note that the following approaches are not intended to replace the classical approach discussed above—they rather constitute an addition to the existing toolkit for characterizing natural vision.

## 3. A complementary approach for characterizing internal models of the world

### (a) Describing internal models

**Figure 2.**
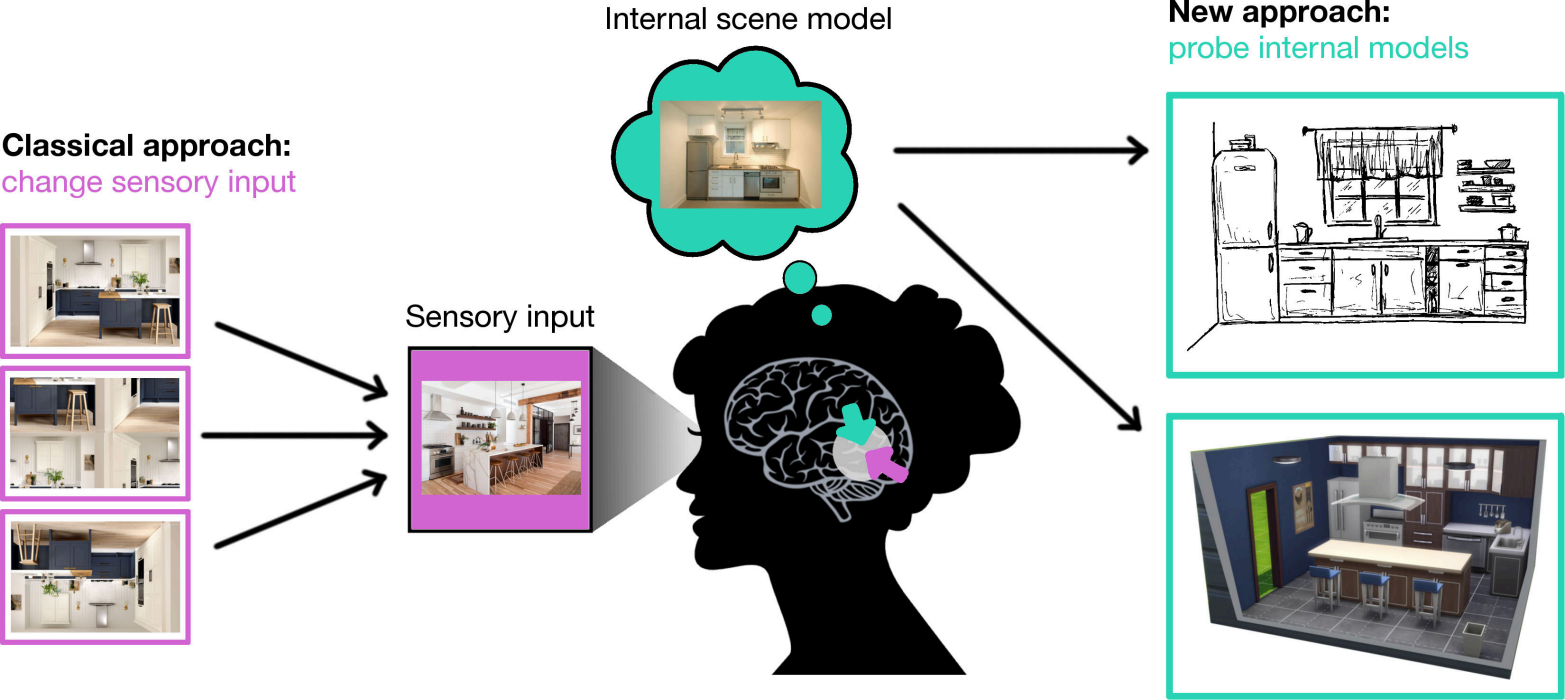
A complementary approach for studying internal models of the world. The classical approach aims at discovering properties of internal models through stimulus manipulation, for instance by manipulating a scene’s global structure. Here, we highlight a complementary approach, in which the contents of internal models are described by observers, for instance through line drawing (where people draw typical versions of scenes) or scene arrangement methods (where people arrange physical or virtual scenes in typical ways). These descriptions can, in turn, be used to derive targeted predictions about processing efficiency for a set of inputs.

Here, we highlight a novel, complementary approach that characterizes individual participants’ internal models of scenes in more unconstrained ways, without prior assumptions about their properties (figure 2). The key idea is to ask participants to provide ‘descriptions’ about the contents of their internal model that can then inform further investigation. By obtaining such descriptions of internal models, we can start to understand how individuals converge and diverge in their conceptions of scene typicality. Moreover, we can in turn use descriptions of internal models to make targeted predictions about processing efficiency for individual scenes in individual observers.

This approach circumvents critical limitations of the classical stimulus manipulation approach. First, researchers do not need to use their intuitions about which features constrain internal models of natural scenes, as they can rather rely on the features emerging from participants’ descriptions. For instance, rather than manipulating specific features based on prior knowledge, such as the object position or overall scene context (figure 1), individual descriptions provide direct access to the features prioritized across participants. Second, multiple interacting feature dimensions can be studied at once, as these will inherently be present in the descriptions. Third, there is no need to artificially create atypical stimuli: the individual typicality of a range of stimuli can be quantified relative to the descriptors. Finally, by focusing on descriptions of internal models in individuals, we can understand to what extent our priors are general or idiosyncratic.

How can we experimentally obtain such descriptions of internal models? We will discuss several methods that enable individual participants to convey their idea of a typical scene exemplar. One strong candidate is drawing, which has proven to be a versatile tool for transforming mental representations into visible descriptions [46]. Beyond that, we discuss scene arrangement, linguistic descriptions and neuroimaging-based techniques. We illustrate these techniques with a range of studies, primarily focusing on the memory and perception literature.

### (b) Line drawings as descriptors of internal models

Line drawings can be seen as functional abstractions of the ways in which we see the world in the sense that they ‘exploit the underlying neural codes of vision’ [47]: when we draw an object or a scene, our drawing tends to focus on what conveys the most essential details of visual images in a form that abstracts away from irrelevant detail. Line drawings of scenes are recognized with virtually identical efficiency as scene photographs [48], probably because they preserve critical information about the curvature and intersection of contours [49,50]. Neuroimaging work has shown that line drawings yield characteristic category-specific neural activation patterns in the high-level visual cortex [50,51]. Beyond theoretical reasons, drawings also offer practical advantages: they are easy to generate and enable the creation of rich scenes in an (almost) unconstrained manner. These qualities render drawings an ideal candidate for the description of internal models. In the following, we highlight how these advantages have been leveraged in clinical and developmental settings, before turning towards research in memory and perception.

#### (i) Line drawings in clinical assessment and developmental research

The use of drawings has a long history in clinical assessment to diagnose and classify visual impairments such as agnosia [52], spatial neglect [53] or different types of neurodegenerative disease [54,55]. Furthermore, systematic differences in drawing tasks have been described for individuals with psychiatric disorders, including autism spectrum disorder [56] and schizophrenia [57]. These two conditions are associated with compromised predictive processing [58,59], suggesting that alterations to drawings in these disorders may be linked to alterations in internal models of the world.

**Figure 3.**
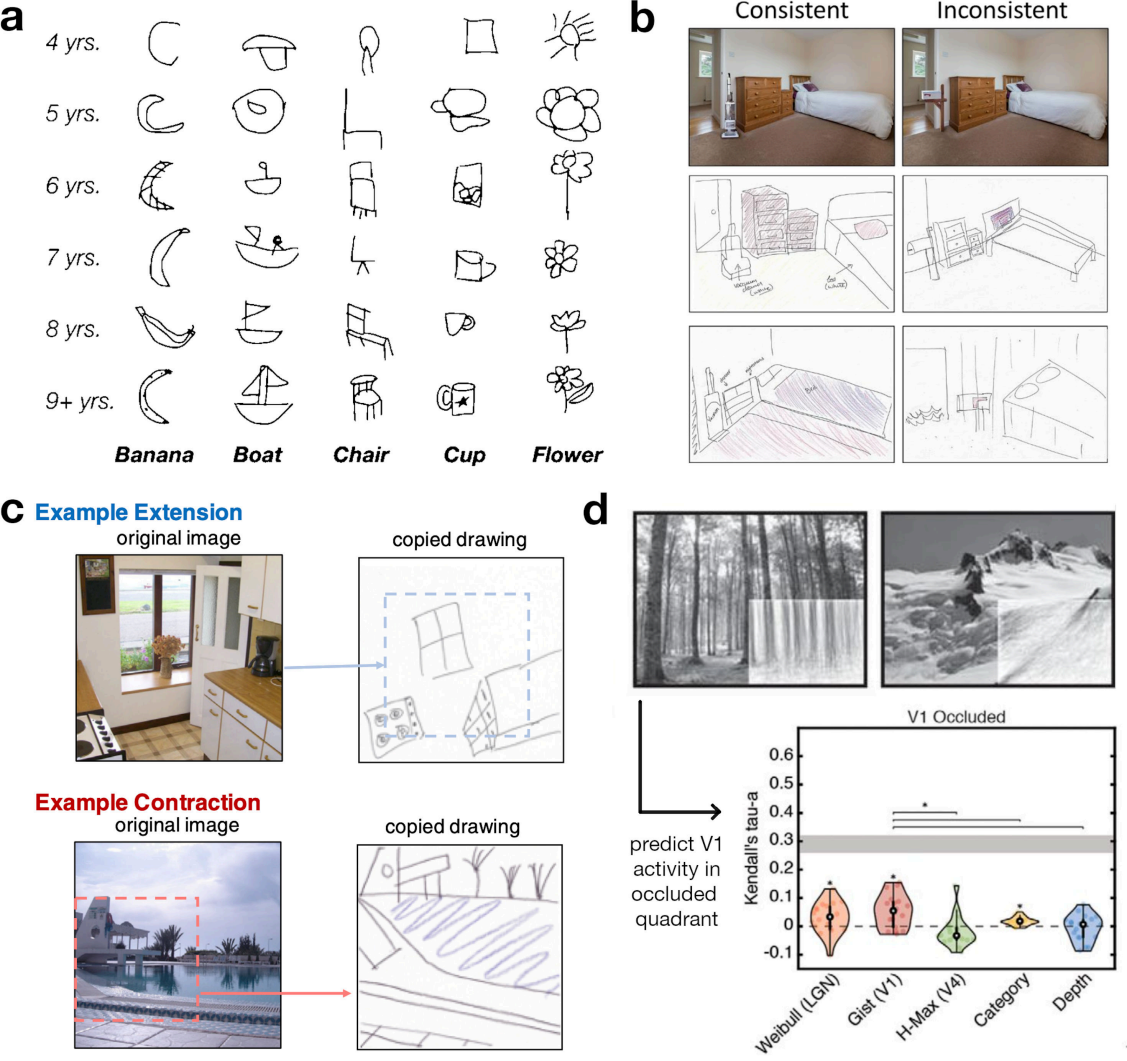
Using drawings to describe representations in development, memory and perception. (a) In developmental research, the use of drawings allows researchers to gain insights into the emergence of detailed visual object representations (figure is reproduced from [62] under a CC BY 4.0 license). (b) In memory research, drawings can be used to quantify memory precision in free recall paradigms. For instance, in scenes with inconsistent objects, more detail about the inconsistent object is recalled, at the expense of recalling details of the scene (figure reproduced from [63]). (c) Using a similar free recall paradigm, Bainbridge & Baker [64] showed that scene boundaries are extended or compressed in memory, depending on the viewpoint and geometry of the original scene. (d) In perception research, drawings were used to probe the cortical filling-in of missing information. Participants’ drawings of what should be present in an occluded quadrant predict neural activation: response patterns in areas of primary visual cortex (V1) that respond to the occluded quadrant are well explained by visual low-level visual features of these drawings [65].

In a similar vein, drawings have proven extremely useful for studying internal models across development. For instance, drawings have been used to assess the emergence of detail in visual representations of objects [60–62]. In recent work, Long *et al*. [61] asked children across different ages to draw various everyday objects, revealing characteristic changes in drawings across development (figure 3a). Interestingly, the content of children’s drawings predicted their ability to recognize visual objects [62], suggesting a link between the mental representation revealed by drawings and the ability of the visual system to process critical object details. Together, these findings indicate that drawings mirror the visual system’s ability to efficiently represent visual inputs.

#### (ii) Line drawings in studying memory

The use of drawings for studying internal representations in healthy adults dates back around a century. Metzger [66] described how participants made systematic drawing errors when copying line drawings under unfavourable conditions such as low contrast, miniature size or peripheral presentation. These reproductions tended to appear more unitary, regular and tightly structured than the originals, suggesting a normalization process towards internal perceptual templates.

**Figure 4.**
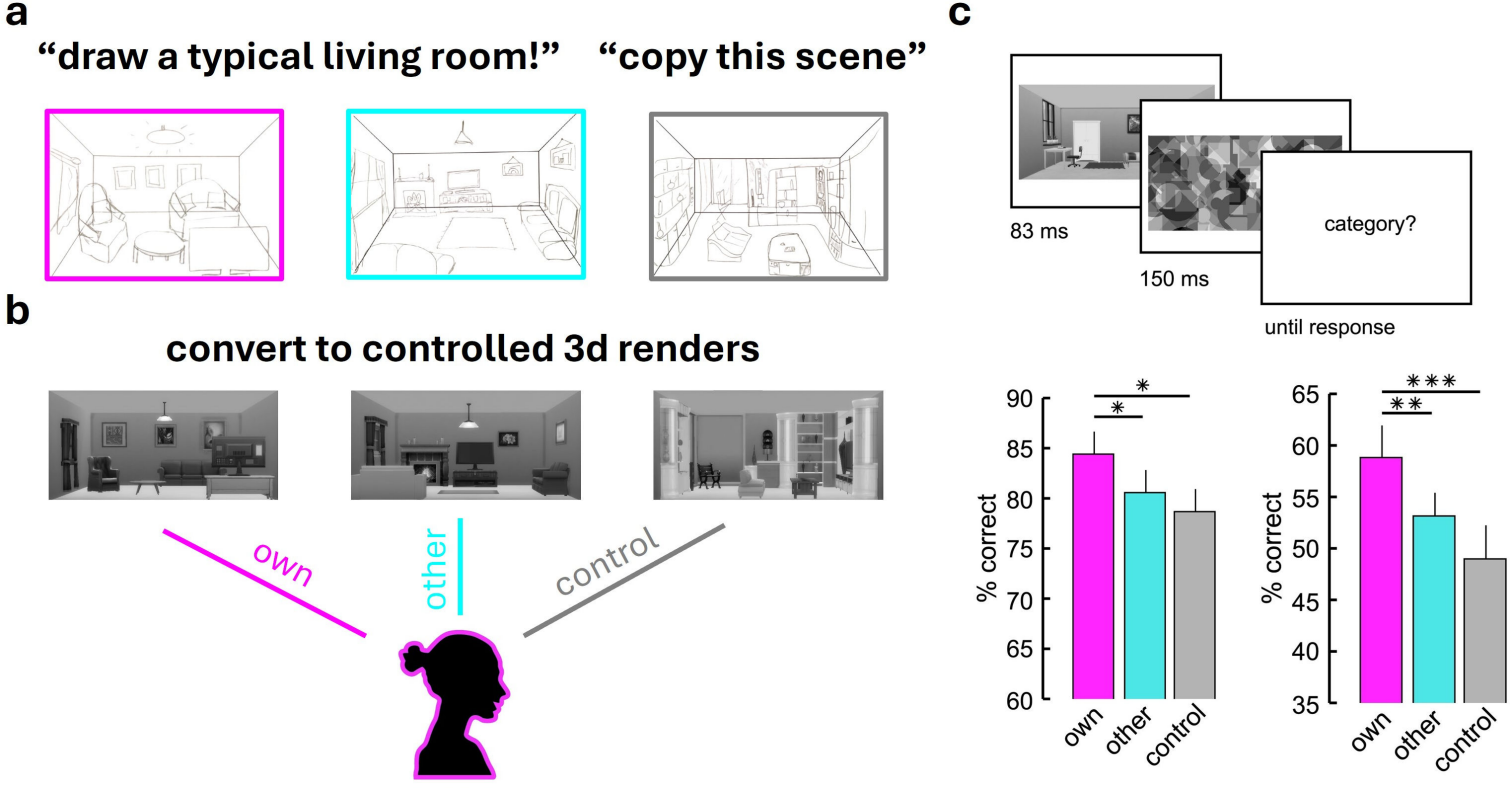
Using drawings to link individual differences in internal models to idiosyncrasies in perception. (a) To assess the contents of internal models for real-world scenes, participants drew typical versions of scene categories (here: living rooms). (b) These drawings were converted to three-dimensional renders to control for visual differences. (c) During the subsequent categorization task, participants categorized briefly presented renders. Critically, they viewed renders based on their own drawings (‘own’ condition), other participants’ drawings (‘other’ condition) or renders created from scenes participants previously copied from a photograph (‘control’ condition, designed to control for drawing-related familiarity effects). Across two experiments with two (left) or six (right) scene categories, participants more accurately categorized renders from the ‘own’ condition than renders from the ‘other’ or ‘control’ conditions, suggesting that similarity to internal models on the individual level modulates scene processing in idiosyncratic ways [68].

More recently, line drawings received renewed attention in the memory literature, enabling unconstrained free-recall tasks on complex scenes [46]. For instance, Bainbridge *et al.* [67] uncovered a surprising amount of detail in memory drawings of scenes and objects. The precision of memory drawings varies as a function of scene typicality: semantically inconsistent objects are remembered more vividly than semantically consistent objects—at the expense of weaker memory for other scene characteristics in the incongruent images [63] (figure 3b). Memory drawings also reveal the degree of boundary extension in natural scene images. In a large-scale analysis, Bainbridge & Baker [64] demonstrated that this effect is ultimately stimulus-dependent, with the tendency for boundaries to be extended for scenes perceived as near and compressed for those perceived as distant (figure 4c), interpreted as a normalization towards a typical viewing distance. In this trade-off, the depth of field plays a critical role, such that a naturalistic depth of field leads to a larger boundary extension than a non-naturalistic depth of field [69]. Together, these findings further illustrate how drawings can help to understand the intricacies of human memory representations.

#### (iii) Line drawings in visual perception

In the study of perception, drawings have been used more sparingly. Yet, they are a promising method for studying predictive processes. In predictive processing frameworks, perception is often described as a generative model in which a percept is constructed by employing the internal world model to infer the most likely cause of sensory input [1]. Drawings offer researchers a tool to access these hypotheses by serving as an extension of the generative process into a visible format.

In line with this idea, drawings have been used to capture representations of invisible but predictable visual content in the visual cortex [65]. In this study, participants viewed natural scene images, in which one quadrant was occluded while functional magnetic resonance imaging (fMRI) activity was recorded from areas of the early visual cortex that exclusively respond to input from the occluded quadrant. Outside the scanner, participants were asked to draw the likely content of the occluded quadrant. Results showed that basic visual features of the drawings predicted scene-specific activations in the unstimulated area of the primary visual cortex (figure 4d). This finding highlights that drawings can be used to capture the content of neural predictions.

More recently, Wang *et al.* [68] used drawings as a readout of individual participants’ internal models of visual scenes (figure 4). Here, participants were asked to draw typical versions of a set of natural scene categories (e.g. kitchens or living rooms). These drawings were converted into standardized three-dimensional renders to control for different drawing abilities and styles. In a subsequent categorization task, participants were more accurate in categorizing renders that were constructed from their *own* drawings (and were thus more similar to their *own* internal models) than in categorizing renders based on *other* participants’ drawings (which were more dissimilar to their *own* internal models). The authors further showed that the similarity to the scene renders based on participants’ own drawings (measured by a deep neural network model) predicted categorization accuracy on other rendered scenes. This result demonstrates how drawings can be used to make personalized predictions about the efficiency of perception—derived from only a single drawing of a typical scene. Complementary EEG work [70] showed that neural representations of scenes that are similar to participants’ drawings (and thus their internal models) are enhanced during perceptual processing unfolding in the initial 250 ms of visual analysis. This suggests a rapid interaction between the visual input and an observer’s internal model during natural vision, underscoring the importance of idiosyncratic representations in scene perception. Although the current results are based on a recognition task, we believe that other behaviours are also shaped by an individual’s internal models. This opens new avenues for research into inter-individual variability in other tasks, for example, how individuals navigate through a scene or search for an object.

However, drawing methods also come with limitations, such as the challenge of objectively quantifying the contents of drawings and handling the substantial inter-subject variability in drawing abilities and style. Variation in drawing expertise has been associated with inter-individual differences in cognitive and perceptual abilities such as visual imagery, shape encoding and detection, as well as the allocation of visual attention and working memory [71–74]. Different approaches to minimizing such potentially confounding factors have been put forward: sufficient sample sizes and suitable control conditions can mitigate variance related to drawing abilities. Further, innovative methods like pen-tracking, computer vision or online crowdsourcing provide objective and reproducible tools for quantifying drawings [46,75].

In sum, drawings have proven a powerful tool for describing internal representations and have advanced our understanding of the precision of visual memory, the development of visual object representation and the perception and neural representations of scenes. Nevertheless, the methodology of drawings comes with limitations. In the following, we discuss three alternative methods for characterizing the contents of internal models, which offer complementary strengths.

### (c) Alternative descriptors of internal models

We highlight three alternative ways of assessing the contents of internal models. The first two approaches (scene arrangement and linguistic descriptions) build on a similar principle as drawings by enabling participants to describe their internal model using physical or virtual objects, as well as language. Finally, we highlight an approach that attempts to directly infer characteristics of subjects’ internal models from brain recordings without relying on overt reports.

#### (i) Scene arrangement

In scene arrangement paradigms, participants create a scene by arranging a set of candidate objects provided by the experimenter. Although such methods often limit participants’ degrees of freedom in describing their internal models (e.g. because of fixed object exemplars available for arrangement), they mitigate the issue of inter-individual variability in drawings.

**Figure 5.**
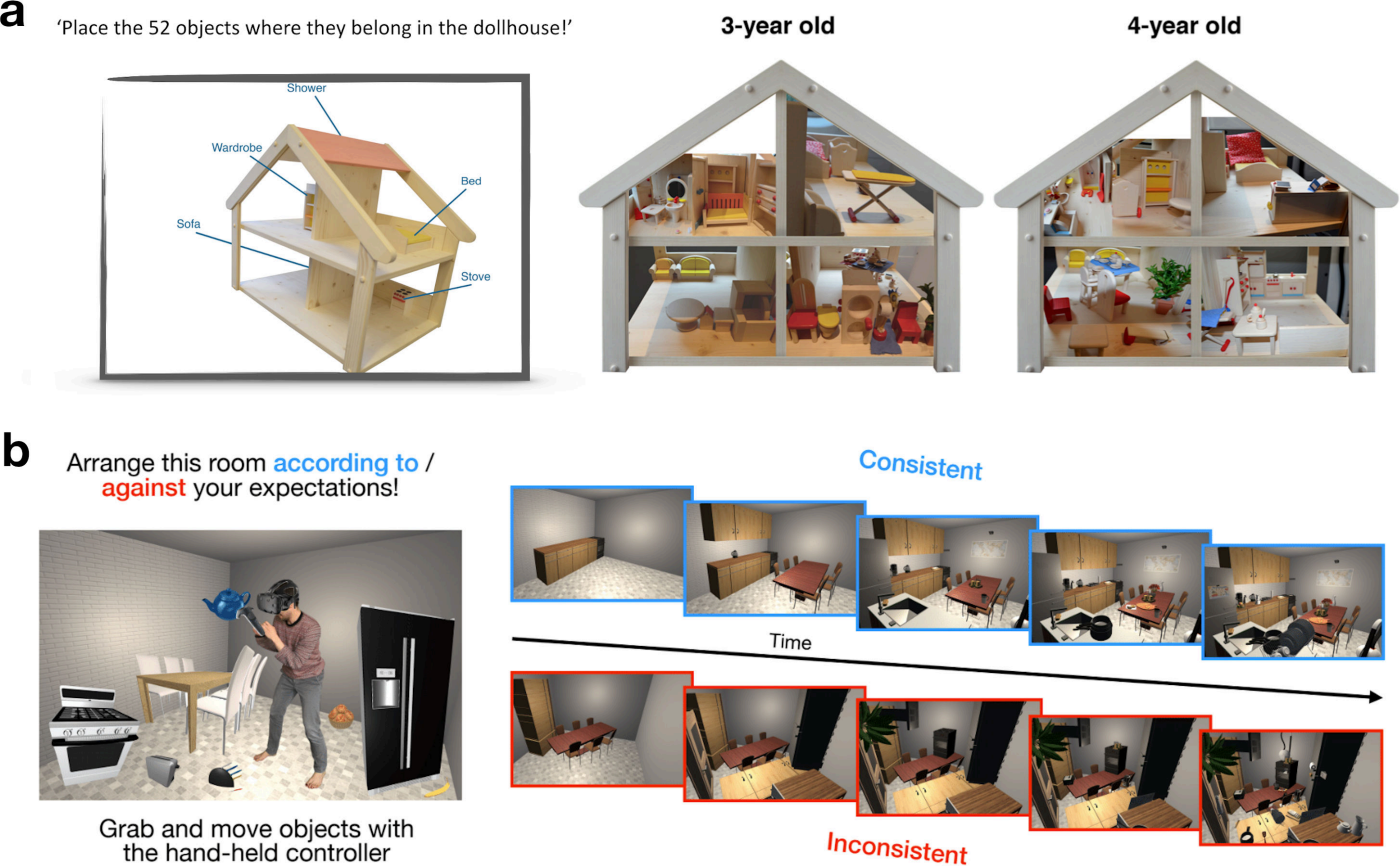
Using explicit scene arrangement to describe internal models. (a) Children of different age groups were asked to arrange a set of miniature objects across a dollhouse. Object arrangements showed that children first appreciate semantic object similarities and only later incorporate the typical spatial organization across groups of objects [77]. (b) Participants arranged objects in a VR environment into typical or atypical configurations. In subsequent search and memory tasks, participants performed better when the task was situated in the scenes constructed in a typical fashion (figure reproduced from [78] under a CC BY 4.0 license).

Scene arrangement tasks can be realized with real physical objects. For example, Öhlschläger & Võ [76] used an arrangement task in a doll house to test how children across different age groups honour semantic relationships (e.g. chairs and tables appear together in a dining room) or spatial regularities (e.g. chairs face the dining table) between objects (figure 5a). Results showed that children as young as 3 years respected semantic relationships but not spatial regularities among multiple related objects, while children over 4 years arranged the objects in semantically and spatially congruent ways. Using the same task, Bahn *et al.* [79] showed that the performance of scene arrangement tasks covaries with language development, suggesting a link between the linguistic concept organization and the visual rules that structure natural scenes.

Such real-world scene arrangement tasks, however, require real objects that need to be moved around in physical space. As an alternative, virtual reality (VR) allows for highly controlled, easily manipulable and interactive environments [80]. Showcasing this potential, Draschkow & Võ [78] asked participants to construct scenes that concurred with their internal models of typical scenes (e.g. placing the objects in a kitchen in a typical fashion) or that violated them (e.g. placing the same objects in an atypical fashion) (figure 5b). In subsequent experiments, participants more successfully searched and memorized scenes arranged in typical ways, compared with scenes arranged in atypical ways. These results show how descriptors of internal models, as captured by explicit scene arrangement, can in turn be used to test perception and memory in environments that are specifically tailored to individual participants’ internal models.

Another promising variant of scene arrangement methods is adjustment procedures, where participants linearly vary scenes along one or more dimensions to match their internal model. Such approaches have, for instance, been used for matching colour expectations [81] or peripheral appearance [82]. For scenes, such adjustments can easily be realized for low-level properties such as contrast or colour. Yet, high-level properties can also be adjusted through continuous scene spaces derived from generative computational models, as for instance implemented in the ‘scene wheel’ [83], which provides seamless continua between individual scene exemplars. Such adjustment methods, however, depend on the experimenter’s choice of dimensions, but may strike a balance between experimental control and participant-driven insights into internal model properties.

#### (ii) Linguistic descriptions

Focusing more directly on conceptual rather than visual attributes of scenes, linguistic descriptions of scenes offer another effective tool that is independent of sensory-motor demands. Such reports are comparably easy to obtain, and they can carry rich semantic detail. Yet, they may be less precise in capturing some of the aspects that drawings convey (e.g. spatial organization).

When asked to describe scene images, our linguistic descriptions are tightly linked to our perception of scenes. For example, atypical scenes are harder to describe after brief exposure compared with typical scenes [84]. Furthermore, linguistic descriptions are constrained by individual differences in the ways we explore scenes [85]: inter-subject similarities in fixation patterns during free viewing can be predicted by inter-subject similarities in subsequent scene descriptions. For instance, participants who mentioned people more often in their descriptions also looked at people more prominently during exploration, and participants who mentioned text more often spent more time looking at text. This finding highlights the potential of linguistic descriptors to capture information about scene representations on the individual level.

Future studies could employ linguistic descriptions to gauge the contents of internal models directly, revealing the conceptual factors that organize our priors. Specifically, similar to the paradigm used by Wang *et al.* [68] wherein participants were asked to draw typical versions of a set of natural scene categories and then tested on categorization for scenes similar or dissimilar to these drawings, participants could alternatively provide linguistic descriptions of what they think a typical exemplar of a specific scene category should look like. The similarity of any given scene image with the linguistic descriptions provided by individual participants could, in turn, be used to predict perceptual efficiency or neural responses for these scenes on the individual level. Alternatively, generative text-to-image models could be used to generate stimulus materials that are in accordance with, or deviate from, individual participants’ linguistic descriptions. However, the outputs of generative models are influenced by the model’s own priors acquired throughout training. The model’s priors may be difficult to disentangle from the participant’s internal model.

#### (iii) Neural quantification of internal models

As an alternative to behavioural reports, the content of participants’ internal models could, in principle, be read out from neural responses. This is an exciting future prospect because brain activity recorded during simple visual tasks or passive fixation is potentially less prone to subjective biases and task-specific demand characteristics.

Studies linking scene typicality to neural responses are the foundation for this idea. Scenes that align with internal models strongly produce more diagnostic, ‘sharpened’ neural responses [86]. This variation in visual responses can be used to derive ‘neural prototypes’ of a given category in individual participants, for instance, by averaging responses to a larger set of exemplars into a prototypical response [87] or by comparing an exemplar’s activity pattern to all other members of that category, following the premise that a typical exemplar shares more attributes with other members of its category than atypical exemplars [88]. However, more work is needed to characterize how typicality is processed in the brain before neuroimaging-based methods can be used to read out a person’s internal model with confidence.

Moreover, neuroimaging-based approaches remain inherently constrained by the experimenter’s selection of stimulus materials, which may not cover natural visual distributions sufficiently and in unbiased ways. To mitigate this constraint, large and diverse stimulus sets that feature thousands of scene instances, such as the Natural Scenes Dataset (NSD; [89]), provide useful benchmarks. A promising direction for future research is to combine neural measures of typicality with free production approaches, such as drawings or scene arrangement, thereby leveraging their complementary strengths.

## 4. Challenges in describing internal models

The methods described above enable researchers to infer the content of internal models with fewer constraints and prior assumptions about their properties than classical approaches. However, these methods also come with their own challenges. We highlight three challenges below and outline what we gain from solving them.

First, behavioural descriptions of internal models, such as drawings or linguistic reports, are subjective reports. Can we assume that such introspective insights are reliable? Introspection is often disregarded as inherently problematic because observers may not be able to reliably characterize their internal representations [90,91]. However, this view has been challenged, most prominently by Gestalt psychologists [92], but also more recently [93,94], with proponents arguing that introspection offers converging information compared with analytic approaches. Further, the quality of introspective insight can be addressed empirically by quantifying whether introspective insights about internal models indeed predict the efficiency of perception. Our review, therefore, does not make the case to replace or overcome classical approaches to studying scene vision—we rather need to combine stimulus manipulation approaches with approaches for describing internal models.

Second, some of the outlined methods require separating informative differences from incidental variance across individuals. This is particularly relevant for drawings, where different drawing styles and abilities [95,96] introduce substantial variation that is not directly related to our internal representations. Moreover, methods like scene arrangement may bias the provided descriptions by offering a limited number of available objects. A careful choice of methods, control conditions, sample size and analysis approach is required to minimize these shortcomings and unfold the complementary strengths of these techniques. Additionally, participants may prioritize some objects over others during production. Reasons for this include biases towards larger and more salient objects (e.g. a sink is more often drawn than a toothbrush) or more scene-diagnostic objects (e.g. a socket is rarely drawn, given it is not diagnostic for a room category). Such biases may yield imperfect descriptors of internal models.

Third, although subjective reports such as drawings offer rich insights into internal models, they may not be ideal to clarify the complexity of features in the internal model. Recent work shows that individual differences in internal models are expressed in complex high-level features coded in late layers of deep neural network models [70], suggesting that complex object- and scene-related features structure our internal models. Yet, low-level properties may prominently feature in internal models, too, and drawings can capture some of such low-level regularities, such as visual density, texture continuity or edge alignment. Other low-level properties, like colour distributions or texture properties, are harder to capture with drawings and may elude investigation with drawing methods.

In addition, most of the methods highlighted here yield single descriptions of participants’ internal models, implying that there is a single, stable internal model for a given scene category. However, internal models may encompass multiple typical scene configurations (e.g. private versus public bathrooms). They may also be shaped by context (e.g. in the form of precision weighting [1]), recently formed associations (e.g. through serial dependence effects [97]) and behavioural goals [98]. This requires experiments that repeatedly quantify the contents of internal models within the same participants, thereby characterizing how internal models change across time and context.

## 5. Conclusion

We showed that classical approaches to characterizing internal models of natural scenes through stimulus manipulation have notable limitations, including an over-reliance on *a priori* assumptions about scene typicality, restricted possibilities for stimulus manipulation, the use of artificial control stimuli and insensitivity to inter-individual differences. We therefore highlight a complementary methodological framework that allows for more unconstrained descriptors of participants’ internal models. One promising method is the use of line drawings, which has recently opened new avenues in the study of visual memory and perception. Overall, we believe that natural vision research greatly benefits from methods with fewer constraints and prior assumptions about the nature of internal models, complementing traditional approaches. Although we focused on characterizing internal models for natural scenes, we believe that approaches targeting the contents of internal models can be very informative in other visual domains. Indeed, drawings have been used for understanding mental generalization in object recognition [99,100], perceptual distortions in peripheral vision [101] and individual differences in mental imagery [102]. Embracing this approach could yield novel insights into how internal models differently shape perception across individuals and across cultural and linguistic contexts, and how alterations of internal models drive changes in visual processing across the lifespan and from health to disease.
